# Imaging comparative analysis of familial and sporadic gout in Chinese men by multijoint ultrasonography

**DOI:** 10.3389/fmed.2024.1477220

**Published:** 2024-11-20

**Authors:** Wen Wen, Liwen Ma, Wantai Dang, Ping Lei, Jing Hu, Jian Liu

**Affiliations:** ^1^Department of Ultrasound, First Affiliated Hospital, Clinical Medical College of Chengdu Medical College, Chengdu, China; ^2^Department of Ultrasonic Medicine, Chengdu Wenjiang District People’s Hospital, Chengdu, China; ^3^Department of Rheumatology, First Affiliated Hospital, Clinical Medical College of Chengdu Medical College, Chengdu, China

**Keywords:** gout, tophi, high-frequency ultrasound, family history, joint

## Abstract

**Objective:**

This study aimed to compare the imaging features of bilateral knees, ankles, and the first metatarsophalangeal joint using high-frequency ultrasonography in male patients with familial and sporadic primary gout and sought to elucidate the relationship between the presence of tophi and various clinical indicators.

**Method:**

Male patients with primary gouty arthritis (GA) in the acute phase presenting to the Department of Rheumatology and Immunology at the First Affiliated Hospital of Chengdu Medical College from November 2020 to June 2022 were enrolled and classified into familial and sporadic gout groups. Comparative analyses of their clinical data and ultrasonographic imaging findings of the knees, ankles, and first metatarsophalangeal joints were performed between the groups. Univariate and multivariate logistic regression analyses, as well as receiver operating characteristic (ROC) analysis, were conducted to determine the effectiveness of significant factors in the prediction of tophi.

**Result:**

In comparison to male patients with sporadic gout, those with familial primary gout exhibited lower age, body mass index, disease duration, and serum uric acid (SUA) levels. However, they demonstrated higher incidences of tophi and bone erosion (54.6% in familial gout vs. 35.1% in sporadic gout, *p* < 0.05; 71.2% in familial gout vs. 48.1% in sporadic gout, *p* < 0.05, respectively), with a greater prevalence of tophi in the right first metatarsophalangeal joint (44.2% in familial gout vs. 32.3% in sporadic gout, *p* < 0.05). Independent risk factors for tophi included family history (OR = 6.712), age (OR = 1.049), disease duration (OR = 1.134), and SUA levels (OR = 1.006). ROC analysis yielded an area under the curve of 0.883 (*p* < 0.05) for predicting joint tophi using these factors.

**Conclusion:**

Male patients with familial primary GA in the acute phase experienced earlier onset, shorter disease duration compared to those with sporadic gout. They also had more affected joints, more frequent and a wider distribution of tophi, especially in the right first metatarsophalangeal joint. Family history, age, disease duration, and SUA levels are predictive of tophi formation.

## Introduction

Gout is a systemic metabolic disorder arising from abnormalities in purine metabolism, leading to the deposition of crystallized monosodium urate (MSU) in the joints and periarticular soft tissues, thereby precipitating acute episodes of gouty arthritis (GA). The global incidence of gout is notably high, with a prevalence rate of 9.2% in men, approximately four times higher than that in women (1). Beyond episodic acute arthritis, gout is associated with chronic joint damage, subcutaneous tophi, and periarticular inflammation. As tophi develop, bone erosion exacerbates, consequently leading to organ failure and impairments in joint movement, which significantly diminish patients’ quality of life (2, 3).

Clinically, gout can be categorized into familial and sporadic forms, based on the presence or absence of a family history of the disease, respectively. Approximately 20% of gout patients are reported to have a familial history of the condition (4). Purine metabolism disorders are prevalent in majority of the patients with familial primary gout and is responsible for the elevation of serum uric acid (SUA) level and formation of tophi through the precipitation of urate crystals (5). Genome-wide association studies (GWAS) for gout have identified approximately 183 loci that influence SUA levels, with 55 of these loci being associated with an increased risk of gout. These identified loci account for approximately 7.7% of the variation in SUA concentrations, indicating a genetic predisposition to the condition (6). Findings from twin studies indicate that the heritability of serum urate ranges from 45 to 73% (7). While the familial aggregation of gout is influenced by both genetic and lifestyle/biological factors, accumulating evidence suggests that the magnitude of familial risk escalates with increasing genetic relatedness. The risk is highest among individuals with multiple affected first-degree relatives, followed by siblings, and then offspring (8).

Over the past two decades, the rapid advancement of High-frequency Ultrasound (HFUS) examination technology has positioned ultrasonography as a primary imaging technique for the assessment of GA, owing to its convenience, comprehensiveness, speed, and efficacy. Furthermore, HFUS is favored as a practical imaging modality for the early detection of MSU crystal deposition, demonstrating superior sensitivity and specificity for the knee joint and the first metatarsophalangeal joint compared to other imaging techniques (9, 10). In 2015, the Outcome Measures in Rheumatology (OMERACT) Ultrasound Working Group established standardized definitions for structural lesions in gout, including the double contour sign, tophi, hyperechoic aggregates, and bone erosion (11). The utility of ultrasonography in the evaluation of GA continues to be substantiated.

Considering the contribution of genetic factors to the intricate pathogenesis of gout, it is plausible that patients with a familial history of the disease are more predisposed to developing intra-articular tophi compared to those without genetic predisposition. However, there has hardly been any reporting on the comparative analysis of tophi between patients with familial and sporadic primary gout. Therefore, this study enrolled 395 male patients diagnosed with primary gout and collected ultrasonographic images of their bilateral knees, ankles, and first metatarsophalangeal joints using high-frequency ultrasound. Additionally, demographic characteristics, chronic comorbidities, and various biochemical indices were recorded. The objective was to compare the imaging features and investigate the relationship between the presence of tophi and various clinical indicators in male patients with familial and sporadic primary gout.

## Methods

### Patients

This study encompassed male patients with primary GA in the acute phase who presented for the first time to the Department of Rheumatology and Immunology at the First Affiliated Hospital of Chengdu Medical College between November 2020 and June 2022. The inclusion criteria were as follows: (1) patients who met the 2015 classification criteria for gout established by the European League Against Rheumatism and the American College of Rheumatology (12); (2) those aged between 18 and 85 years; and (3) those who had not received any treatments related to the reduction of uric acid, blood pressure, or glucose within the preceding 3 months. The exclusion criteria for the study were delineated as follows: (1) patients diagnosed with rheumatoid arthritis, reactive arthritis, psoriatic arthritis, spinal arthritis, or other forms of inflammatory arthritis; (2) individuals with secondary gout attributable to tumor radiotherapy, chemotherapy, hematologic disorders, renal disease, or the use of specific medications; and (3) participants with incomplete data records. Informed consent was obtained from all participants, and the study received approval from the Medical Ethics Committee of the First Affiliated Hospital of Chengdu Medical College (Approval No. 2019CYFYHEC-BA-34).

### Identification of gout family history

In our study, familial gout is defined as the presence of at least one case of gout among first-degree or second-degree relatives, in addition to the index case (defined as an individual diagnosed with primary gout). Specifically, family members within three generations of the first affected case are classified as “exposed” from the date of diagnosis of the index case. The second diagnosed family member is defined as the first “familial case,” and subsequent cases are categorized accordingly. Only individuals with identifiable biological parents were enrolled in the study. The diagnostic information for these familial cases is obtained from the hospital electronic medical record system, which clearly includes the family relationship information for all enrolled patients. The diagnostic information includes the age, sex, time of gout diagnosis, and the hospital where the diagnosis was made for familial cases.

### Clinical data

Clinical data were collected, encompassing variables such as age, body mass index (BMI), disease duration, history of cigarette smoking and alcohol consumption, and medical history, which primarily included hypertension and type 2 diabetes mellitus (T2DM). Disease duration was defined as the interval between the initial onset of gout and the date on which ultrasonography was conducted. Blood samples were obtained from each patient on the day of the ultrasonographic examination for routine hematological assessments, including SUA, erythrocyte sedimentation rate (ESR), serum creatinine (Scr), alanine aminotransferase (ALT), aspartate aminotransferase (AST), serum total cholesterol (TC), triglyceride (TG), C-reactive protein (CRP), glucose (GLU), cystatin C (CysC), and other relevant biomarkers.

### US examination

The bilateral knees, ankles, and first metatarsophalangeal joints of the enrolled patients were evaluated using ultrasonography scanner (GE LOGIQ E9 Diagnostic Ultrasound Scanner, GE Healthcare, Waukesha, WI, USA) with a high-frequency wide-band linear array probe (ML9-15). The examinations were conducted by a sonographer with 10 years of experience, who was blinded to the patients’ clinical data. The presence and distribution of tophi and bone erosion were documented by two independent sonographers who reviewed all images separately. A positive ultrasonographic sign was confirmed only when there was concordance between the two reviewers.

**Figure 1:**
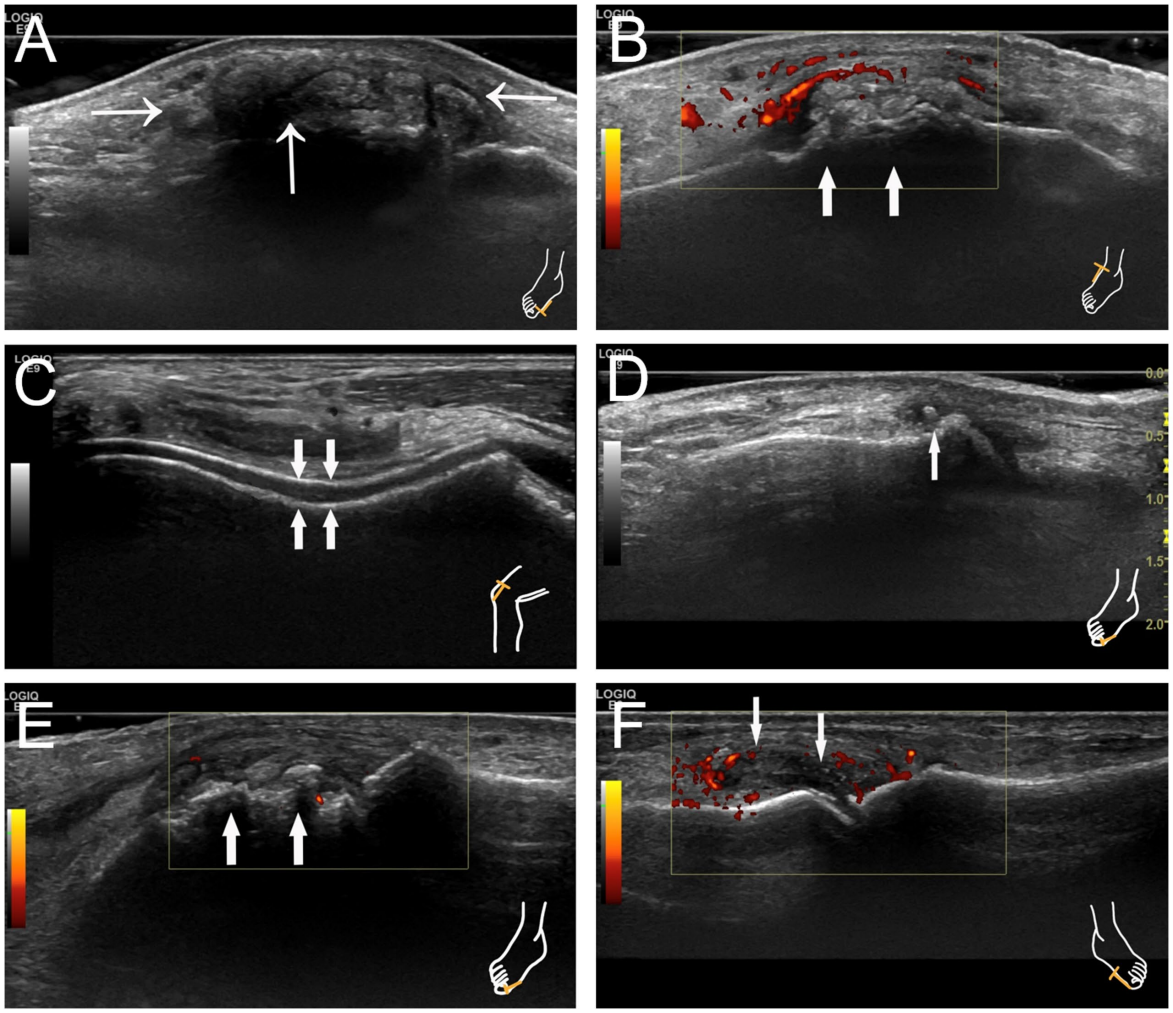
Characteristic images of joint ultrasonography in patients with gout. **(A)** Tophus with posterior acoustic shadow at the right first metatarsophalangeal joint indicated by thin arrows. **(B)** Tophus with hyperechoic echo at the right knee indicated by thick arrows. **(C)** The double-track sign at the right knee indicated by thick arrows. **(D)** Hyperechoic aggregates at the right first metatarsophalangeal joint indicated by a thin arrow. **(E)** Bone erosion at the right first metatarsophalangeal joint indicated by thick arrows. **(F)** Synovial hyperplasia at the left first metatarsophalangeal joint with synovitis indicated by thin arrows.

The following US signs were assessed: (1) Tophi, defined as nodules composed of inhomogeneous material, exhibiting either hyperechoic or hypoechoic echoes, and occasionally surrounded by a small anechoic rim or accompanied by a posterior acoustic shadow (Figures 1A,B). (2) The double-track sign, characterized by a continuous or intermittent band of abnormal hyperechoic areas situated at the periphery of the articular cartilage surface, independent of the angle of insonation, and presenting either a regular or irregular appearance (Figure 1C). (3) Aggregates, identified as hyperechoic clusters located within the joint, synovial membrane, or adjacent tendons (Figure 1D). (4) Bone erosion is defined as a discontinuity of the bone surface (visible in two perpendicular planes) that may be accompanied by a cortical extra-articular defect (Figure 1E). (5) Synovial hyperplasia is characterized by hypoechoic echo located in the joint capsule and the edge convex into the capsule, with a thickness of more than 2 mm. Sometimes it may be accompanied by punctate strong echo deposition, which is narrowed without being pressurized by the probe. Abundant blood flow signal can be seen in CDFI during acute inflammatory attack (Figure 1F).

### Statistical analysis

Continuous variables were presented as mean ± standard deviation if normally distributed or median (lower quartile, upper quartile) otherwise. Categorical variables were presented as numbers (percentages). Student’s *t*-test, Mann–Whitney rank-sum tests, or chi-squared tests were used, as necessary, to compare the relevant variables. To determine the effectiveness of significant factors in the prediction of tophi, univariate and multivariate logistic regression analyses and receiver operating characteristic (ROC) analysis were conducted. The statistical software SPSS 21.0 (IBM Corporation, Armonk, NY, USA) was used for all statistical analyses, and statistical significance was defined as a *p* value of <0.05.

## Results

### Comparison of the general clinical data between familial primary gout and sporadic primary gout groups

A total of 395 male participants were included in the study, comprising 104 individuals (26.3%) with familial primary gout and 291 individuals with sporadic primary gout. There were no cases of twins among the patients. The mean age of the patients was 46.67 ± 15.30 years. The baseline characteristics of familial primary gout group and sporadic primary gout group were summarized in Table 1. Compared to the sporadic primary gout group, patients with familial primary gout were younger, had a lower BMI, and exhibited a shorter duration of disease. Notably, the levels of SUA, CysC, ALT, CRP, and ESR in patients with familial primary gout were significantly lower than those in patients with sporadic primary gout, with the differences being statistically significant. However, there were no differences between the two groups in terms of histories smoking, alcohol use, hypertension, T2DM and levels of Scr, AST, TG, TC, and GLU.

**Table 1:** Baseline characteristics of familial gout group and sporadic gout group.

Variables	Familial gout (*n* = 104)	Sporadic gout (*n* = 291)	*P* value
Age (years)	41.76 ± 13.33	48.42 ± 15.59	**<0.001**
BMI (kg/m^2^)	25.81 ± 3.83	26.69 ± 3.50	**0.031**
Course of disease (year)	2.85 (0.42, 9.85)	4.35 (1.40, 10.40)	**0.004**
Hypertension (case)	23 (22.10%)	65 (22.30%)	1.000
T2DM (case)	6 (5.80%)	28 (9.60%)	0.309
Smoking (case)	45 (43.30%)	132 (45.40%)	0.732
Alcohol use (case)	57 (54.80%)	151 (51.90%)	0.648
SUA (μmol/L)	510.01 ± 112.16	527.19 ± 105.05	**0.047**
Scr (μmol/L)	82.85 (75.40, 95.15)	85.35 (73.80, 96.40)	0.238
CysC(mg/L)	1.02 (0.90, 1.18)	1.07 (0.95, 1.27)	**0.036**
ALT (U/L)	33.00 (21.00, 53.00)	40.00 (24.75, 66.25)	**0.034**
AST (U/L)	26.00 (20.00, 36.50)	24.00 (18.00, 33.00)	0.158
TG (mmol/L)	2.05 (1.42, 2.75)	1.89 (1.33, 2.75)	0.120
TC (mmol/L)	4.73 (4.25, 5.23)	4.63 (3.97, 5.25)	0.747
GLU (mmol/L)	5.48 (5.16, 6.20)	5.34 (4.81, 6.17)	0.235
CRP (mg/L)	2.00 (1.00, 16.00)	6.00 (2.00, 26.00)	**0.004**
ESR (mm/h)	7.00 (3.00, 17.00)	12.00 (6.00, 31.00)	**0.001**

BMI: Body mass index; T2DM: Type 2 diabetes mellitus; SUA: Serum uric acid; Scr: Serum creatinine; CysC: Cystatin C; ALT: Alanine aminotransferase; AST: Aspartate aminotransferase; TG: Triglyceride; TC: Serum total cholesterol; GLU:Glucose; CRP:C-reactive protein; ESR: Erythrocyte sedimentation rate. Bold values refer to statistically significant *p* values.

### Comparison of ultrasonography between familial primary gout and sporadic primary gout groups

US features, including tophi, bone erosion, double contour sign, hyperechoic aggregates, and synovial hyperplasia of bilateral knees, ankles, and the first metatarsophalangeal joint, were collected from 395 male patients diagnosed with either familial primary gout or sporadic primary gout (Table 2). The analysis indicated that among the patients with familial gout, 74 (71.2%) exhibited tophi and 50 (48.1%) exhibited bone erosion. In contrast, among those with sporadic primary gout, 159 (54.6%) exhibited tophi and 102 (35.1%) exhibited bone erosion. The differences in the prevalence of tophi and bone erosion between the two groups were found to be statistically significant. Other US features did not statistically differ.

**Table 2:** Comparison of ultrasound features between familial gout group and sporadic gout group.

Ultrasound features	Familial gout (*n* = 104)	Sporadic gout (*n* = 291)	*P* value
Tophi	74 (71.2%)	159 (54.6%)	**0.004**
Bone erosion	50 (48.1%)	102 (35.1%)	**0.025**
Double contour sign	66 (63.5%)	171 (58.8%)	0.417
Hyperechoic aggregates	78 (75.0%)	221 (75.9%)	0.894
Synovial hyperplasia	59 (56.7%)	177 (60.8%)	0.486

Bold values refer to statistically significant *p* values.

**Figure 2:**
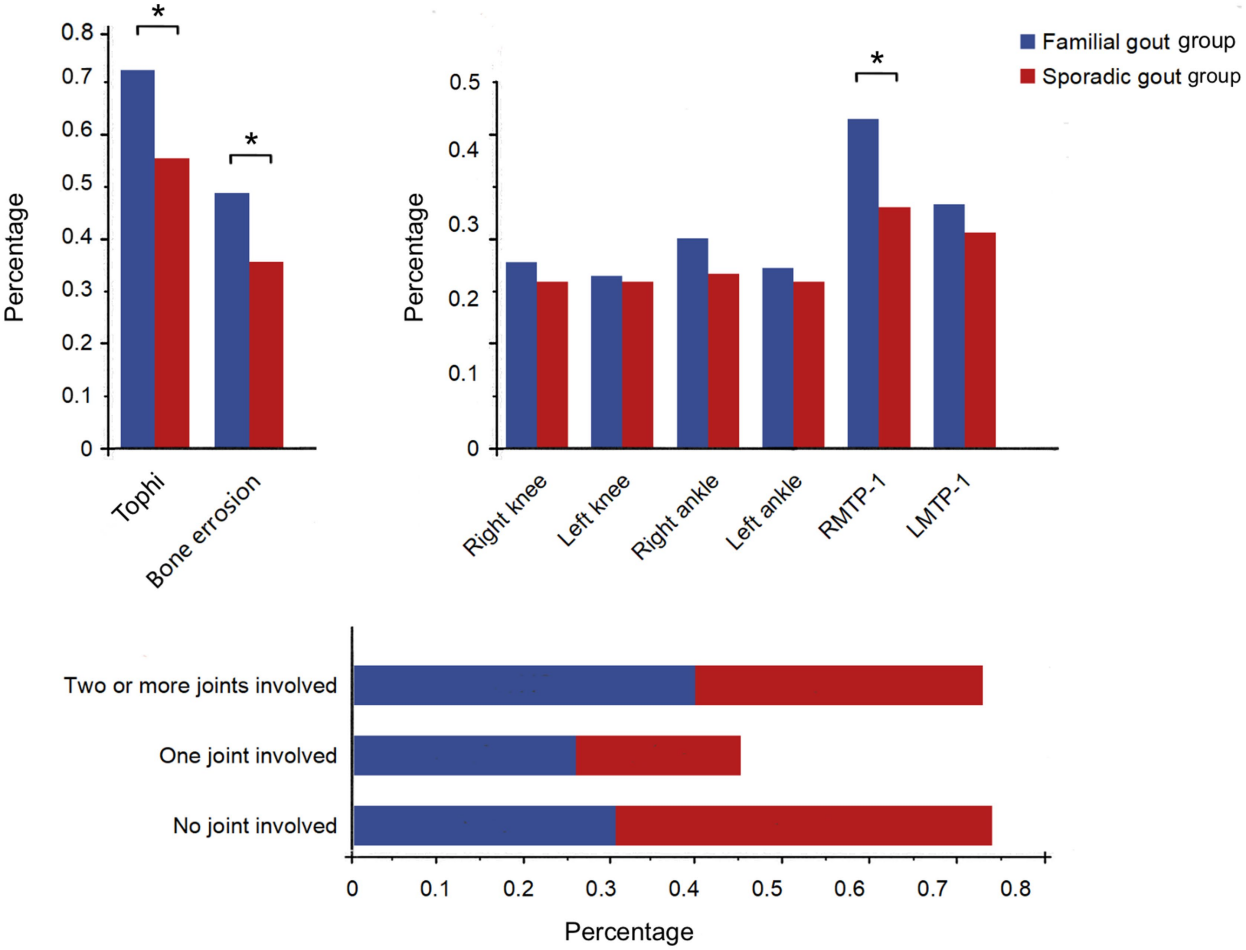
Comparison of affected joint sites and numbers between familial gout group and sporadic gout group. RMTP-1: Right first metatarsophalangeal joint; LMTP-1: Left first metatarsophalangeal joint. * *p* < 0.05.

Furthermore, our findings indicate that familial primary gout patients exhibited a total of 192 (30.8%) affected joints, a number significantly higher than that observed in sporadic primary gout patients (25.3%). Both groups demonstrated instances of multiple joint involvement: within the familial primary gout cohort, 28 cases (26.9%) involved a single joint, while 43 cases (41.3%) involved two or more joints. In contrast, the sporadic primary gout group had 58 cases (19.9%) with single joint involvement and 101 cases (34.7%) with involvement of two or more joints. Significant differences were observed between the two groups concerning the number of joints involved, with a higher number of multiple joint involvements (*p* < 0.05) (Figure 2). Furthermore, the right first metatarsophalangeal joint was predominantly affected in both groups, however, the proportion of patients with familial primary involvement was significantly higher compared to those with sporadic cases [46 familial cases (44.2%) vs. 94 sporadic cases (32.3%), *p* < 0.05] (Supplementary Table S1 and Figure 2).

### Regression analysis of the presence of joint tophi and clinical characteristics

Through a comparative analysis of the two groups, it was observed that tophi were more frequently detected in patients with familial primary gout compared to those with sporadic primary gout. Consequently, we further investigated the correlations between tophi and various clinical variables, including age, BMI, disease duration, family history of gout, among others, as well as additional laboratory indicators. Univariate logistic regression analysis revealed that the presence of tophi was significantly associated with age, disease duration, hypertension, alcohol consumption, family history of gout, SUA, Scr, CysC, ALT, CRP, and ESR levels in male patients with primary gout (Table 3). In multivariate-adjusted models, family history of gout (OR = 6.712, 95% CI = 3.133–14.376), age (OR = 1.049, 95% CI = 1.025–1.075), disease duration (OR = 1.134, 95% CI = 1.057–1.216), SUA (OR = 1.006, 95% CI = 1.006–1.012) and ESR (OR = 1.017, 95% CI = 1.001–1.033), were independent risk factors for tophi formation in male patients with primary gout (Table 3).

**Table 3:** Univariate and multivariate analysis for the formation of joint tophi in male patients with primary gout.

Variables	Univariate analysis			Multivariate analysis		
	OR	95%CI	*P* value	OR	95%CI	*P* value
Age	1.062	1.045–1.080	**<0.001**	1.049	1.025–1.075	**<0.001**
BMI	0.983	0.930–1.039	0.55			
Course of disease	1.26	1.187–1.339	**<0.001**	1.134	1.057–1.216	**<0.001**
Family history	2.048	1.263–3.319	**0.004**	6.712	3.133–14.376	**<0.001**
Hypertension	2.338	1.382–3.956	**0.002**			
T2DM	2.043	0.927–4.502	0.076			
Smoking	0.983	0.657–1.471	0.933			
Alcohol use	1.543	1.031–2.309	**0.035**	1.251	0.709–2.207	0.440
SUA	1.008	1.006–1.011	**<0.001**	1.009	1.006–1.012	**0.046**
Scr	1.009	1.000–1.018	**0.044**	0.996	0.979–1.014	0.651
CysC	3.343	1.737–6.434	**<0.001**	0.954	0.293–3.104	0.938
ALT	0.998	0.993–1.003	0.390			
AST	1.000	0.987–1.013	0.978			
TG	0.998	0.887–1.111	0.973			
TC	0.979	0.893–1.075	0.661			
GLU	1.053	0.938–1.181	0.383			
CRP	1.008	1.000–1.016	0.061			
ESR	1.023	1.011–1.035	**<0.001**	1.017	1.001–1.033	**0.035**

BMI: Body mass index; T2DM: Type 2 diabetes mellitus; SUA: Serum uric acid; Scr: Serum creatinine; CysC: Cystatin C; ALT: Alanine aminotransferase; AST: Aspartate aminotransferase; TG: Triglyceride; TC: Serum total cholesterol; GLU:Glucose; CRP: C-reactive protein; ESR: Erythrocyte sedimentation rate; OR: Odds ratio; CI: Confidence intervals. Bold values refer to statistically significant *p* values.

### The capability of gout family history identifying joint tophi in male patients with primary gout

**Figure 3:**
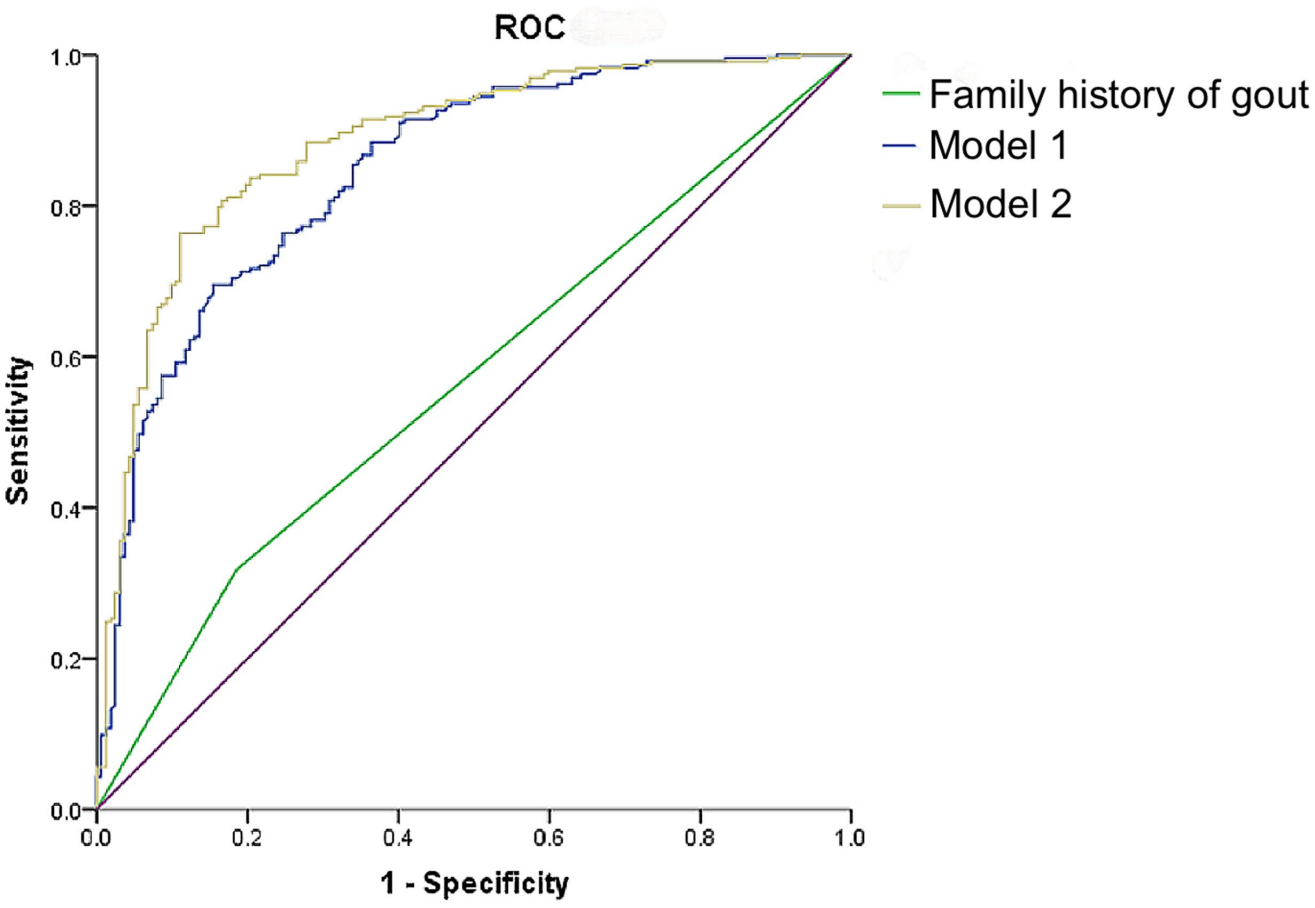
ROC curves of family history of gout, age, course of disease, and SUA level identifying the occurrence of tophi in male patients with primary gout. Model 1: Family history of gout + age + course of disease; Model 2: Family history of gout + age + course of disease + SUA.

To further examine the diagnostic performance of family history in identifying the presence of tophi among patients with primary gout, ROC curves were generated and analyzed in conjunction with regression analysis. Figure 3 presents the ROC curves for predictors of tophi presence in these patients, with the area under the curve (AUC) being 0.562 when considering family history alone (*p* < 0.05). Based on the results of multivariate logistic regression analysis, the capability of combined indicators identifying the presence of joint tophi in patients with primary gout was explored as well. As demonstrated in Supplementary Table S2, the AUC was 0.847 (*p* < 0.05) when a family history of gout was combined with age and disease duration. This AUC increased to 0.883 when SUA levels were also included in the model. Taking both sensitivity and specificity into account, the combination of family history of gout, age, disease duration and SUA provided a robust discriminative value for identifying joint tophi in male patients with primary gout.

## Discussion

In this cross-sectional study comprising 395 male participants, our findings indicate that individuals with familial primary GA in the acute phase experience an earlier age of onset, a shorter duration for tophi formation, a higher prevalence of tophi and bone erosion, an increased likelihood of tophi formation in the right first metatarsophalangeal joint, and a greater number of affected joints compared to those with sporadic primary gout. After adjusting for potential confounding variables, a family history of gout emerged as an independent risk factor for tophi formation in the joints. Furthermore, the ROC curve analysis indicated that a family history of gout, when combined with age disease duration and SUA, was highly predictive of tophi formation in men with primary gout.

Advancements in imaging techniques have enhanced the diagnostic efficacy of high-frequency US and multijoint examinations for detecting tophi and bone erosion in the superficial facet joints and tendons of extremities, particularly the first metatarsophalangeal joint (13). The presence of tophi and bone erosion serves as an indicator of the severity of advanced gout in patients, whereas early gout is characterized solely by urate deposition in the joints (14). Consequently, it is imperative to conduct joint US examinations in patients suspected of having tophus to accurately assess the severity of the disease. Furthermore, the examination may facilitate early intervention and uric acid-lowering therapy, thereby preventing further disease progression and improving patient prognosis (15).

In comparison to patients with sporadic gout, those with familial primary gout exhibit a younger age of onset, lower SUA levels, and a shorter disease duration. Additionally, risk factors for tophi formation include age, family history, and disease duration. We hypothesize two potential reasons for these observations. First, the age of onset for gout in both groups is lower than it was a decade ago (16). With the rapid advancement of medical technology and the increased understanding of gout, a greater number of patients are seeking hospital care promptly upon experiencing the sudden onset of painful, swollen, hot, and erythematous small joints in their extremities. This trend has enhanced the accuracy of early gout diagnosis and has consequently led to a gradual reduction in the misdiagnosis of gout as either a sprain or rheumatoid arthritis. Furthermore, this study found that the onset age of familial gout was, on average, approximately 7 years younger than that of sporadic gout. Previous studies (17) have demonstrated that the early onset of hyperuricemia and urate deposition, leading to acute gouty arthritis in familial patients, is attributable to purine metabolic disorders. These metabolic abnormalities are also responsible for the shorter disease duration observed in patients with familial gout compared to those with sporadic gout. Additionally, inherited tissue factors that facilitate the deposition of MSU crystals in tissues or joints at relatively low serum urate levels may contribute to the lower serum urate levels observed in patients with familial primary gout compared to those with sporadic gout. Subsequently, during a gout flare, the renal excretion rate is elevated, which facilitates the excretion of SUA, thereby normalizing SUA levels (15). Furthermore, due to the deposition of uric acid crystals and the formation of tophi in the joints, patients with familial gout exhibit lower SUA levels compared to those without tophi formation.

In this study, the detection rates of tophi and bone erosion exhibited variability between patients with familial and sporadic gout. Specifically, the detection rate of tophi was higher among patients with familial gout, a finding that contradicts the observations reported in a previous study (18). This discrepancy may be attributable to the present study’s exclusive inclusion of male patients with gout or the potential impact of standard treatment protocols in preventing recurrent inflammation. Similar to the findings of Sun et al. (19), the first metatarsophalangeal joint was most frequently involved in the formation of tophi in both groups, likely due to the propensity for urate deposition at sites of increased mechanical load (20). However, this study revealed a discrepancy in the detection of tophi in the right first metatarsophalangeal joint between the two groups. The presence of tophi in the right first metatarsophalangeal joint was specifically analyzed because all participants in this study were right-handed. During physical activity, the inward pressure results in the first metatarsal bone being the primary load-bearing joint. According to the findings of this study, patients with familial primary tophi exhibit a higher propensity for deposits in multiple joints, often involving two or more joints, and demonstrate a greater prevalence of bone erosion compared to those with sporadic primary gout. The study by Wu et al. (14) elucidated that the presence of tophi is significantly associated with bone erosion; however, the risk of bone erosion escalates with the number of tophi rather than their size. Consequently, it is recommended that a comprehensive multi-joint ultrasound scan be conducted promptly upon the detection of tophi in patients with familial gout to confirm the extent of tophi involvement.

This study also found that family history emerged as a significant risk factor for joint tophi formation in patients with primary gout, as well as for the quantity of tophi deposited in the joints. Prior research has indicated potential associations between tophi formation and genes such as hypoxanthine phosphoribosyl transferase, TGF-β1, GLUT-9, and ABCG2 (21, 22). Despite the congenital nature of most enzyme defects, predominantly affecting males, genetic heterogeneity suggests that the incidence of tophi may vary across different populations. This variability underscores the need for further investigation and comprehensive analysis of affected patients. Previous study (23) by our group have found that family history of gout is a valuable indicator for discriminating grade ≥ 2 left ventricular (LV) diastolic insufficiency in patients with primary gout. Additionally, a combination of family history and SUA level in patients with gout had a better identification performance. We conclude that early clinical identification and intervention for LV diastolic insufficiency can be facilitated by inquiring about a family history of gout in patients diagnosed with the condition. The pathogenesis of gout is influenced by genetic mechanisms. With the rapid advancements in genetic research related to gout, it is now possible to predict the occurrence of gout at the genetic level and tailor personalized medication regimens accordingly. Consequently, incorporating data on family history into the diagnostic and therapeutic assessment of gout can enhance the precision and effectiveness of treatment strategies for patients.

In alignment with the findings of Gao et al. (16), this study identified elevated SUA levels and a family history of gout as significant predictors of gout. Although the interplay between genetic and environmental factors is complex, dietary structure appears to have a minimal effect on SUA levels (24). Previous research has established alcohol consumption as an independent risk factor for the development of tophi (21). Consistent with these prior studies, our univariate correlation analysis also demonstrated that alcohol consumption is a significant risk factor for tophi formation. Although previous research has established a correlation between TG and TC levels and the formation of tophi in patients, our study did not find significant evidence supporting this relationship. Given that the presence of tophi is associated with the uric acid-lowering treatment regimen in gout patients, we subsequently developed a ROC curve to predict the occurrence of tophi in individuals with primary gout. Our findings indicate that family history, along with patient age, disease duration and SUA level exhibit high predictive efficiency for the presence of tophi. Consequently, in patients with gout who present these characteristics, ultrasonographic examination should be considered to accurately detect tophi, thereby facilitating precise diagnosis and treatment.

It has to be admitted that there are some unavoidable limitations in the current study. Firstly, this study is a cross-sectional, single-center investigation with a relatively modest sample size and the analysis was limited to male patients only. Secondly, the diagnosis of gout among relatives primarily relies on medical records, which may introduce potential inaccuracies. This reliance on historical data can lead to underreporting or misclassification of familial gout cases. In the future we plan to implement a more comprehensive approach that includes direct interviews with family members and the use of standardized assessment tools such as genetic testing as a means to corroborate the diagnosis of gout in familial cases. Thirdly, additional factors, including medication history and purine intake history, have yet to be comprehensively examined. Fourthly, the incorporation of emerging technologies, such as contrast-enhanced ultrasonography, shear wave elastographic imaging, and firefly technology, is necessary. Future research should consider the integrated application of multiple imaging techniques.

In conclusion, male patients with familial primary GA in the acute phase had a younger age of onset and shorter disease duration and showed a higher incidence of tophi, with a higher number of joints involved and greater distribution of tophi in the right first metatarsophalangeal joint than those with sporadic primary gout. Family history of gout, age, prolonged duration of the disease, and elevated plasma SUA and ESR level were risk factors for tophi formation in male patients with primary gout. Additionally, family history of gout, along with their age, course of disease, and SUA level, may be a reliable predictor of the development of tophi in those with primary gout. Therefore, for male patients with a family history of gout, it is advisable to consider a joint ultrasound examination at an early stage, along with timely intervention, to potentially mitigate the progression of the disease.

## Data Availability

The raw data supporting the conclusions of this article will be made available by the authors, without undue reservation.
