# Supplementary material for: Imaging comparative analysis of familial and sporadic gout in Chinese men by multijoint ultrasonography
Source: Front Med (Lausanne). 2024 Nov 20;11:1477220. doi: 10.3389/fmed.2024.1477220 (PMC11614634; doi:10.3389/fmed.2024.1477220)
Supplement: Supplementary file 1 [file Table_1.docx]

| **Supplementary Table 1 Comparison of the affected joint sites between**  **familial gout group and sporadic gout group** | | | |
| --- | --- | --- | --- |
| Joint | Familial gout  (n=104) | Sporadic gout  (n=291) | *P* value |
| Right knee joint | 26（25.0%） | 65（22.3%） | 0.589 |
| Left knee joint | 24（23.1%） | 65（22.3%） | 0.892 |
| Right ankle joint | 29（28.2%） | 68（23.4%） | 0.353 |
| Left ankle joint | 27（24.2%） | 65（22.3%） | 0.499 |
| Right first metatarsophalangeal joint | 46（44.2%） | 94（32.3%） | **0.032** |
| Left first metatarsophalangeal joint | 34（32.7%） | 84（28.9%） | 0.457 |

Note: Bold values refer to statistically significant *P* values.

| **Supplementary Table 2 ROC analysis for identifying the occurrence of joint tophi in male patients with primary gout** | | | | | |
| --- | --- | --- | --- | --- | --- |
| Variables | AUC | Sensitivity | Specificity | 95%CI | *P* value |
| Family history of gout | 0.566 |  |  | 0.509-0.623 | **0.025** |
| Model 1 | 0.847 | 0.695 | 0.846 | 0.808-0.886 | **<0.001** |
| Model 2 | 0.883 | 0.764 | 0.889 | 0.849-0.917 | **<0.001** |
| Note: AUC: Area under the curve; CI: Confidence intervals; SUA: Serum uric acid  Model 1: Family history of gout+age+course of disease; Model 2: Family history of gout+age+course of disease+SUA. Bold values refer to statistically significant *P* values. | | | | | |
